# Reduced vasorin enhances angiotensin II signaling within the aging arterial wall

**DOI:** 10.18632/oncotarget.25499

**Published:** 2018-06-05

**Authors:** Gianfranco Pintus, Roberta Giordo, Yushi Wang, Wanqu Zhu, Soo Hyuk Kim, Li Zhang, Leng Ni, Jing Zhang, Richard Telljohann, Kimberly R. McGraw, Robert E. Monticone, Chloe Ferris, Lijuan Liu, Mingyi Wang, Edward G. Lakatta

**Affiliations:** ^1^ Laboratory of Cardiovascular Science, National Institute on Aging, National Institutes of Health, Biomedical Research Center (BRC), Baltimore, MD, USA; ^2^ Biomedical Research Center, Qatar University, Doha, Qatar; ^3^ Department of Cardiology, The First Hospital of Jilin University, Changchun, China; ^4^ Department of Cardiology, Nanfang Hospital, Southern Medical University, Guangzhou, China; ^5^ Department of Vascular Surgery, Peking Union Medical College Hospital, Chinese Academy of Medical Sciences & Peking Union Medical College, Beijing, China

**Keywords:** aging, arterial remodeling, VSMC, vasorin, collagen

## Abstract

The glycosylated protein vasorin physically interacts with the transforming growth factor-beta1 (TGF-β1) and functionally attenuates its fibrogenic signaling in the vascular smooth muscle cells (VSMCs) of the arterial wall. Angiotensin II (Ang II) amplifies TGF-β1 activation in the VSMCs of the arterial wall with aging. In this study, we hypothesized that a reduced expression of the protein vasorin plays a contributory role in magnifying Ang II-associated fibrogenic signaling in the VSMCs of the arterial wall with aging. The current study shows that vasorin mRNA and protein expression were significantly decreased both in aortic wall and VSMCs from old (30 mo) vs. young (8 mo) FXBN rats. Exposing young VSMCs to Ang II reduced vasorin protein expression to the levels of old untreated cells while treating old VSMCs with the Ang II type AT1 receptor antagonist Losartan upregulated vasorin protein expression up to the levels of young. The physical interaction between vasorin and TGF-β1 was significantly decreased in old vs. young VSMCs. Further, exposing young VSMCs to Ang II increased the levels of matrix metalloproteinase type II (MMP-2) activation and TGF-β1 downstream molecules p-SMAD-2/3 and collagen type I production up to the levels of old untreated VSMCs, and these effects were substantially inhibited by overexpressing vasorin. Administration of Ang II to young rats (8 mo) for 28 days via an osmotic minipump markedly reduced the expression of vasorin. Importantly, vasorin protein was effectively cleaved by activated MMP-2 both *in vitro* and *in vivo*. Administration of the MMP inhibitor, PD 166793, for 6 mo to young adult (18 mo) via a daily gavage markedly increased levels of vasorin in the aortic wall. Thus, reduced vasorin amplifies Ang II profibrotic signaling via an activation of MMP-2 in VSMCs within the aging arterial wall.

## INTRODUCTION

Collagen deposition and infiltration of medial vascular smooth muscle cells (VSMCs) into the intima are microscopic characteristics of arterial aging and angioplasty after injury [1–3]. Consequent collagen build-up and intimal VSMCs cellularity result from increased angiotensin II (Ang II) signaling, activation of matrix metalloproteinase type II (MMP-2), and transforming growth factor-beta 1 (TGF-β1) in the aged arterial wall [4, 5].

The Ang II peptide and its receptor AT1 are upregulated in arterial walls with aging, and this phenomenon is closely associated with an enhancement of both sympathetic nerve activity and cyclic mechanical stress [1]. The sympathetic neurotransmitter norepinephrine and its alpha-receptor expression are upregulated in the arterial walls with aging [6–10], contributing to an increase of the Ang II protein abundance and upregulation of the AT1 receptor [4]. The age-associated elevation of pulse pressure increases the arterial cyclic mechanical strain force and subsequently promotes the expression of both Ang II protein and AT1 receptor signaling [11–13]. Importantly, Ang II is also a potent activator of both TGF-β1 and MMP-2 [5].

Vasorin, also known as anti-tissue necrosis factor alpha (TNFα)-induced apoptosis (ATiA) or slit-like 2 (slit2) protein, is a classic type I membrane protein, including 10 tandem arrays of a leucine-rich repeat motif, an epidermal growth factor-like motif, and a fibronectin type III-like motif at the extracellular domain, which is highly glycosylated *in vivo* [2, 14, 15]. This extracellular cell surface glycoprotein, predominantly derived from VSMCs, is a substrate of MMP-2 and is developmentally regulated [16–18]. Notably, vasorin, by physically binding to TGF-β1, functionally limits TGF-β1 downstream signaling such as SMAD-2/3 phosphorylation and collagen production via a blockade of access to its receptor, TGF β receptor type I & II, on the surface of VSMCs [2]. During an acute injury, the amount of vasorin decreases while the amount of TGF-β1 increases, contributing to an imbalance of the vasorin / TGF-β1extracellular protein ratio, which greatly modulates arterial fibrotic remodeling [2, 15].

In the current study, we hypothesize that a reduced expression of vasorin protein, due to its cleavage by MMP-2, amplifies the Ang II/ TGF-β1 fibrogenic signaling in the arterial wall with aging. Indeed, the present *in vivo* and *in vitro* studies, for the first time, documents that aging decreases the expression of vasorin, mainly due to an increase in its cleavage mediated by MMP-2, which consequently amplifies Ang II/TGF-β1 fibrogenic signaling and VSMC invasiveness with advancing age. In contrast, the upregulation of vasorin protein, as well as prevention of its cleavage, substantially delays fibrogenesis and VSMC invasiveness in the arterial wall with aging. Thus, vasorin appears to be a potent novel modulator of Ang II signaling and is a molecular target to retard the fibroplasia of arterial aging.

## RESULTS

### Vasorin expression in arterial walls and VSMCs

To determine the effect of aging on the expression of vasorin in the arterial wall or VSMCs, thoracic aortae were harvested from 8-mo-old young (8 mo) and 30-month-old (30 mo) FXBN rats and VSMCs isolated.

**Figure 1:**
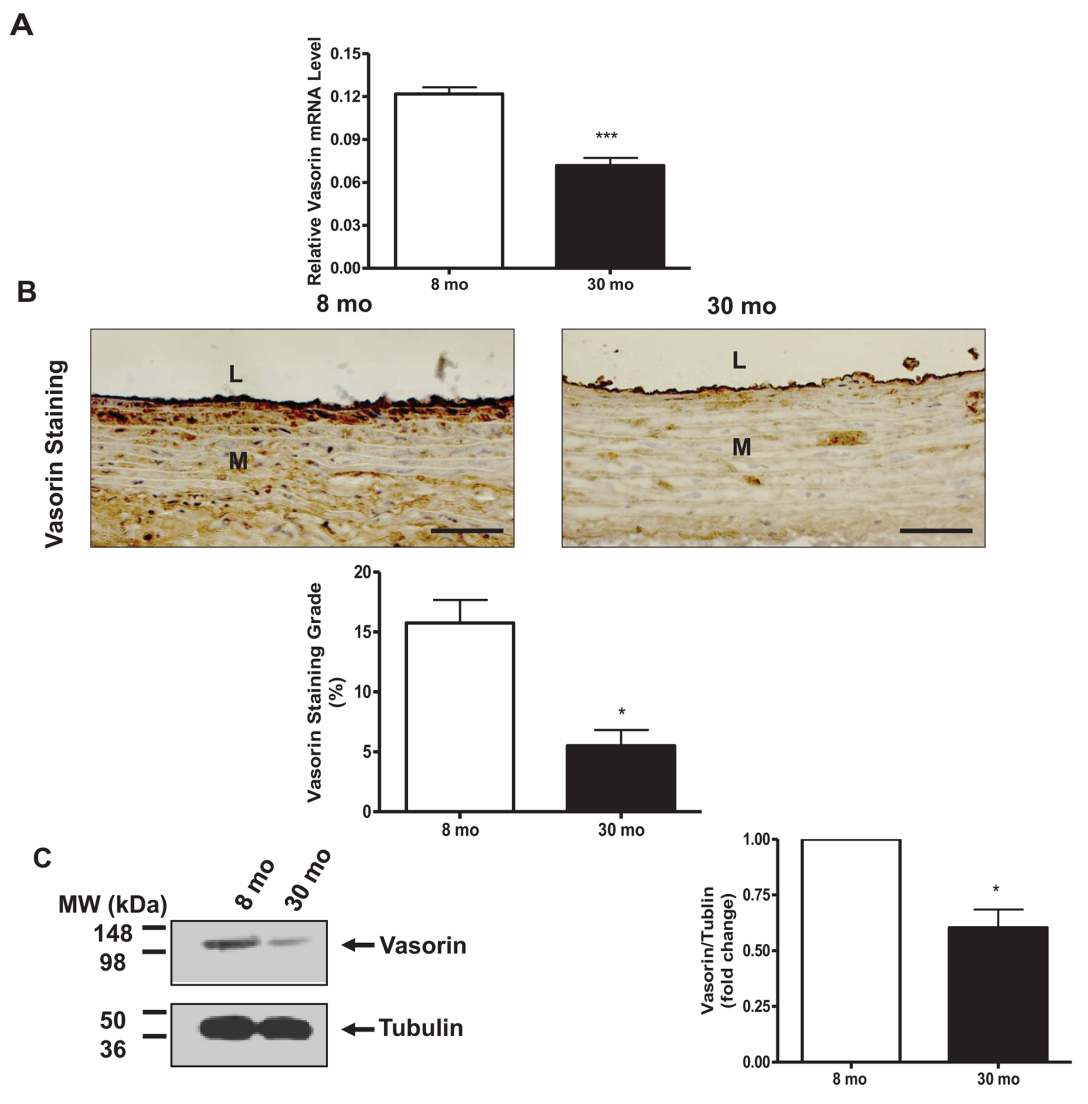
Vasorin expression decreases in arterial walls with aging **(A)** Aortic vasorin mRNA determined by RT-PCR. Data shown as mean ± SEM (n= 4 rats/group). *T*-test, ^***^= p<0.001. **(B)** Immunohistostaining of vasorin (brown color, 400X) in aortic walls. All micrographs were taken at the same exposure time. Data of staining vasorin area/arterial area ratio (%, lower panel) shown as mean ± SEM (n= 4 rats/group). *T*-test, ^*^ =p<0.05. L=lumen; M=media. Bar scale, 10 μm. **(C)** Representative western blots of aortic vasorin. Data (right panel) shown as mean ± SEM (n= 3 rats/group). Each column represents a normalized ratio (fold-changes) to tubulin and to 8 mo. *T*-test, ^*^=p<0.05.

RT-PCR showed that vasorin mRNA levels were markedly decreased in old vs. young rat aortae (Figure 1A). Immunohistostaining of rat aortic walls demonstrated that vasorin protein signal was decreased (∼3-fold) in old vs. young rat aortae (Figure 1B). Western blotting of homogenous rat aortic protein further demonstrated that the expression levels of the vasorin protein were significantly downregulated in old vs. young rats (Figure 1C).

**Figure 2:**
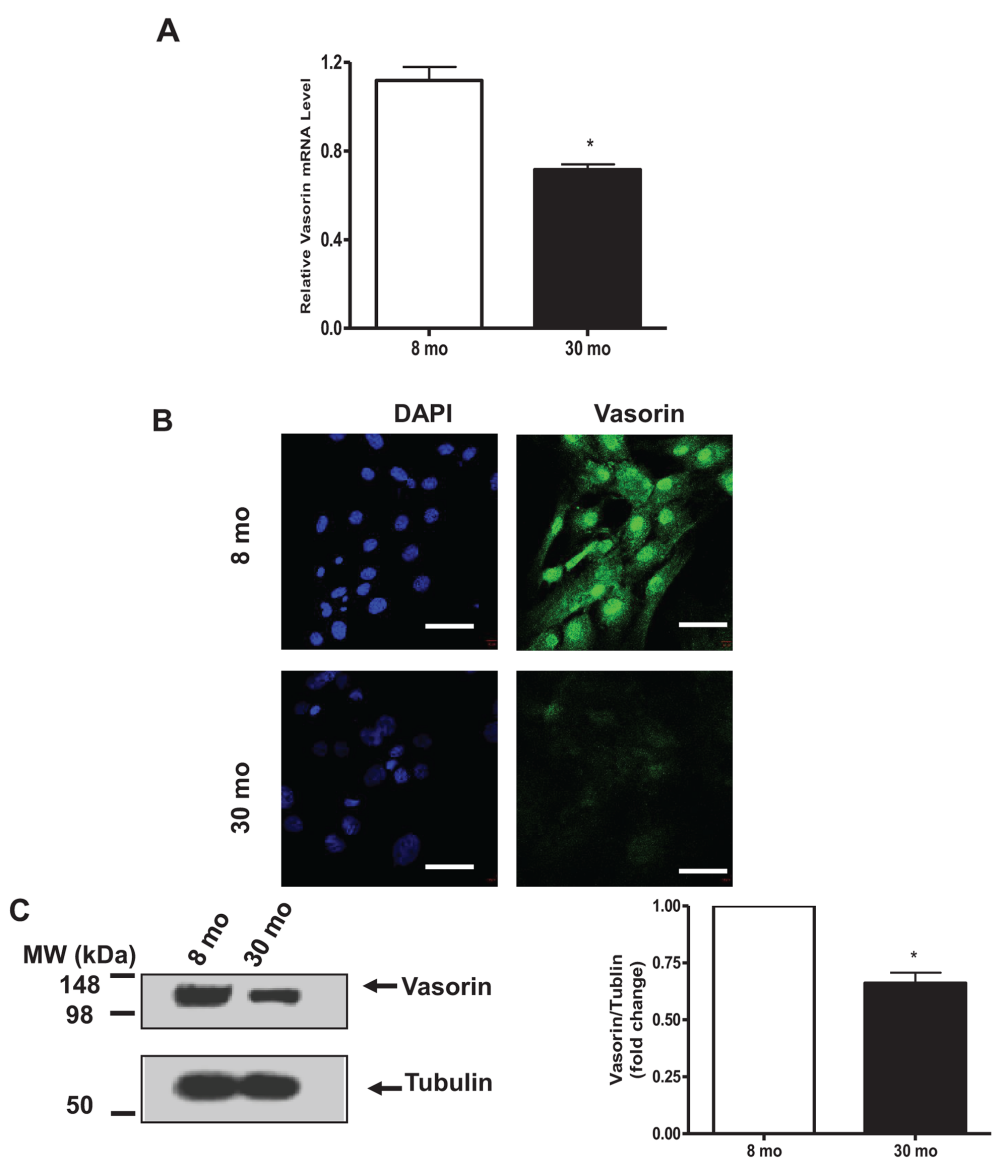
Vasorin expression decreases in VSMCs with aging **(A)** VSMC vasorinm RNA determined by RT-PCR. Data shown as mean *±* SEM (n= 6 rats/group). *T*-test, ^*^=p<0.05. **(B)** Representative immunohistostaining (X400) of vasorin (green) and nuclei (blue) in VSMCs captured by confocal microscopy. All micrographs were taken at the same exposure time. Bar scale, 20 μm. **(C)** Representative western blots of VSMC vasorin. Data (right panel) shown as mean *±* SEM (n=3 independent experiments from n=3 rats/group). Each column represents a normalized ratio (fold-change) to tubulin and to 8 mo. *T*-test, ^*^=p<0.05.

Similarly, vasorin mRNA abundance was significantly decreased in cultured VSMCs isolated from old vs. young rat aortae (Figure 2A); likewise, vasorin immunostaining signal was diminished in old vs. young cells (Figure 2B); and vasorin protein levels determined by immunoblotting were also significantly decreased in old vs. young rat VSMCs (Figure 2C).

### Vasorin expression and Ang II signaling in arterial walls or VSMCs

Ang II increases activation of MMP-2 in the arterial wall and VSMCs with aging [4]. Vasorin is a substrate of activated MMP-2 [17, 18]. Therefore, we next explored the relationships between increased Ang II signaling, vasorin expression and its cleavage in the arterial wall or cultured VSMCs with aging.

**Figure 3:**
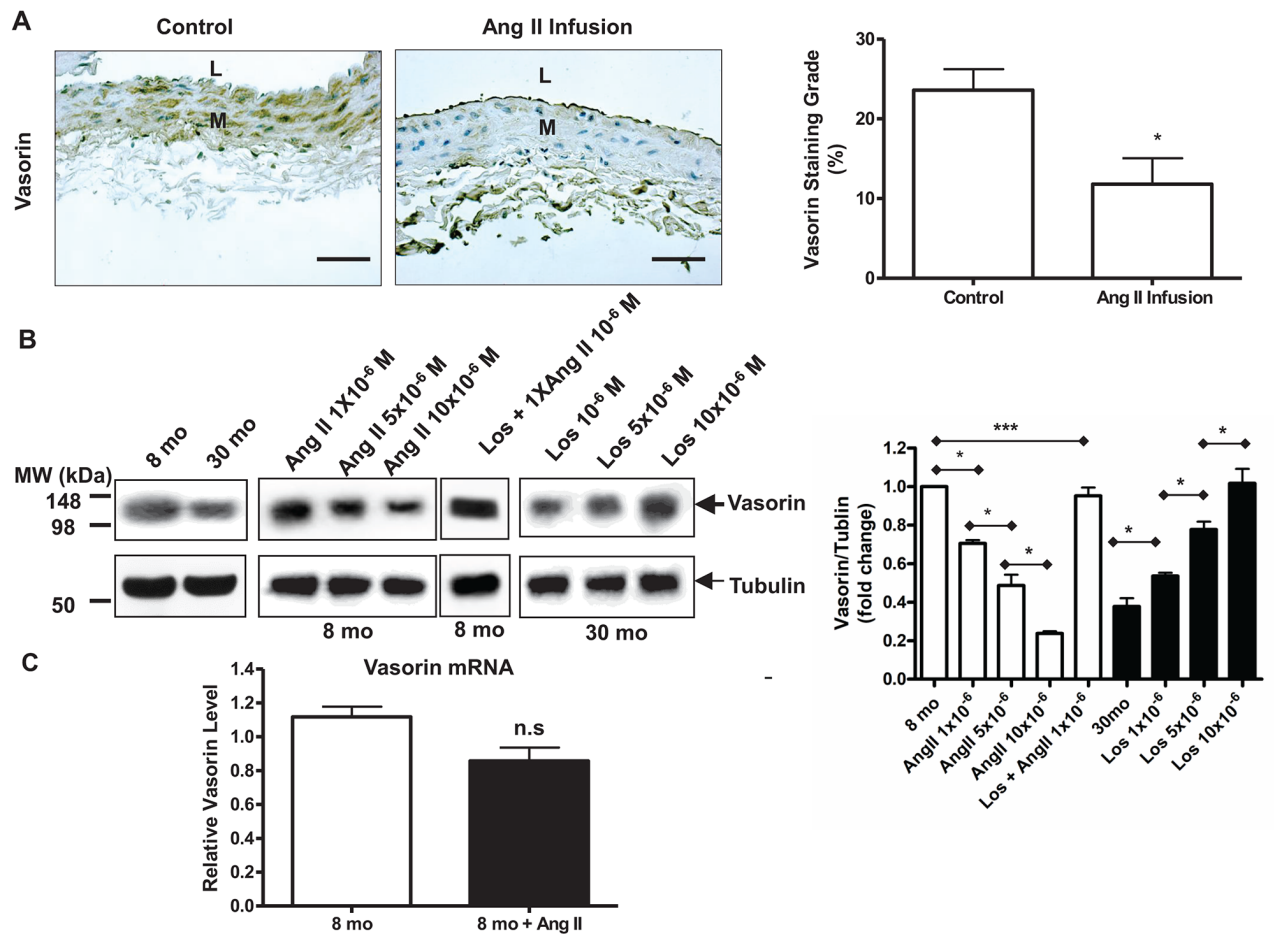
Age decreases vasorin expression in aortic wall or VSMCs via Ang II signaling **(A)** Representative immunohistostaining of vasorin (brown, X400) of carotid arteries harvested from young rats infused with either Ang II (Ang II infusion) or vehicle saline animals (control). All micrographs were taken at the same exposure time. Data of relative vasorin staining area (%) (right panel) shown as mean ± SEM (n= 5 rats/group). *T*-test, ^*^=p<0.05. L=lumen; M=media. Bar scale, 10 μm. **(B)** Representative western blots of VSMC vasorin. Cells have been pre-treated for 1 hour with Los, and then exposed for 24 hours to a medium containing both Ang II and Los. Data (right panel) shown as mean ± SEM (n=4 independent experiments from n=4 rats/group). One-way ANOVA followed Bonferroni post hoc test, ^*^p<0.05, ^**^=p<0.01; and ^***^=p<0.001. Ang II = Angiotensin II; Los=Losartan.; and M=Molar concentration. **(C)** VSMC vasorin mRNA determined by RT-PCR. Cells have been treated for 24 hours in medium containing Ang II. Data shown as mean ± SEM (n= 6 rats/group). *T*-test, n.s = no significant.

Arterial specimens from a prior Ang II infusion experiment were utilized in the following data set [4]. We used immunohistostaining and demonstrated that the administration of Ang II to 8-mo-old young rats (8 mo) for 1 month (Ang II infusion) markedly decreased the expression of vasorin protein in the aortic wall compared to vehicle animals (control) (Figure 3A), which is consistent with the increase of activated MMP-2 described previously [4]. The exposure of young VSMCs to Ang II dose-dependently reduced the expression of vasorin protein down to levels resembling that of untreated old cell (Figure 3B). Importantly, this effect was abolished by the Ang II AT1 receptor antagonist, Losartan (Los) (Figure 3B). Furthermore, treating old VSMCs with Los dose-dependently increased the expression of vasorin protein up to the levels observed in untreated young cells (Figure 3B). Unexpectedly, the treatment of VSMCs with Ang II did not significantly alter the mRNA levels of vasorin as observed for its protein (Figure 3C). These findings strongly suggest that the levels of vasorin protein may be modified by Ang II signaling-associated post-translational modifications, such as the MMP-2-mediating its cleavage.

**Figure 4:**
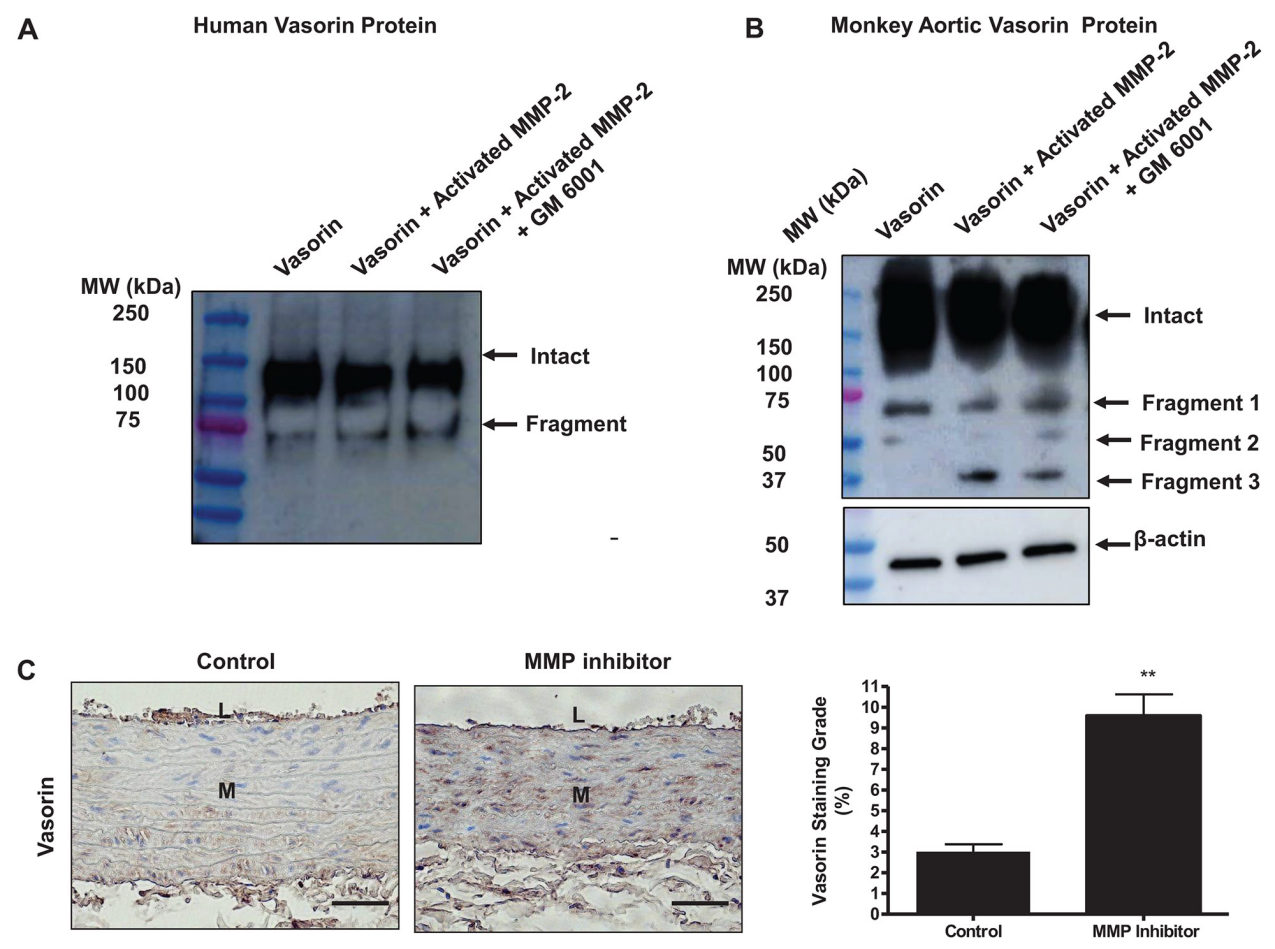
Vasorin is an MMP-2 substrate **(A)** Representative western blots of vasorin showing that the purified human vasorin was cleaved by the activated MMP-2. Recombinant human vasorin (400 ng/ml) was incubated in a developing buffer containing activated MMP-2 (50 nM) with or without an MMP inhibitor GM6001 (20 nM) for 24 hours at 37°C (n= 4 independent experiments). Western blots were probed with vasorin antibody, which detected the intact vasorin (∼100 kDa) and a soluble fragment (∼75 kDa). **(B)** Representative western blots of vasorin showing that the monkey aortic homogenate containing vasorin was cleaved step-wisely by MMP-2. Monkey aortic lysates (25 μg/ml) were incubated in a developing buffer containing activated MMP-2 (50 nM) with or without an MMP inhibitor GM6001 (20 nM) for 24 hours at 37°C (n= 3 independent experiments from 3 monkeys). Western blots were probed with vasorin antibody, which detected the intact vasorin (∼100 kDa) and three soluble fragments (∼75 kDa, 50 kDa, and 37 kDa) showing intact arterial vasorin was processed by activated MMP-2. **(C)** Representative immunohistostaining of vasorin (brown, X400) of aortae harvested from adult young rats administrated with either the MMP inhibitor PD166793 (MMP inhibitor) or vehicle 0.1% dimethyl sulfoxide animals (control). All micrographs were taken at the same exposure time. Data of relative vasorin staining area (%) (right panel) shown as mean ± SEM (n= 5 rats/group). *T*-test, ^**^=p<0.01. L=lumen; M=media. Bar scale, 10 μm.

Indeed, the current *in vitro* and *in vivo* observations indicated that activated MMP-2 has a high capacity to cleave both recombinant human and monkey aortic vasorin protein, which were evidently blocked by the MMP inhibitor, GM 6001 (Figure 4A & 4B). Again, we used specimens from a prior study [5], demonstrating that 6-months administration of the MMP inhibitor, PD 166793, to 18-mo-old young adult rats, markedly increased the level of vasorin (∼3-fold) in the aortic wall (MMP inhibitor) compared to vehicle animals (control) (Figure 4C). Importantly, Ang II or aging induced decrease of vasorin protein abundance was restored by MMP inhibitor, GM6001, treatment (Supplementary Figure 2). In addition, we tested whether a well-known vasorin cleavage proteinase, the disintegrin metalloproteinase 17 (ADM17), may also play a contributory role in the decrease of vasorin protein in the arterial wall with aging [16, 19–22]. Unexpectedly, the abundance of ADAM17 protein was unaltered in VSMCs with aging (Supplementary Figure 1).

Thus, the current *in vivo* and *in vitro* studies demonstrate the reduced levels of vasorin in aging VSMCs and arterial wall are mainly due to its cleavage by MMP-2.

### The physical interaction of vasorin with TGF-β1 acts like an AT1 antagonist and modifies Ang II downstream fibrogenic signaling

**Figure 5:**
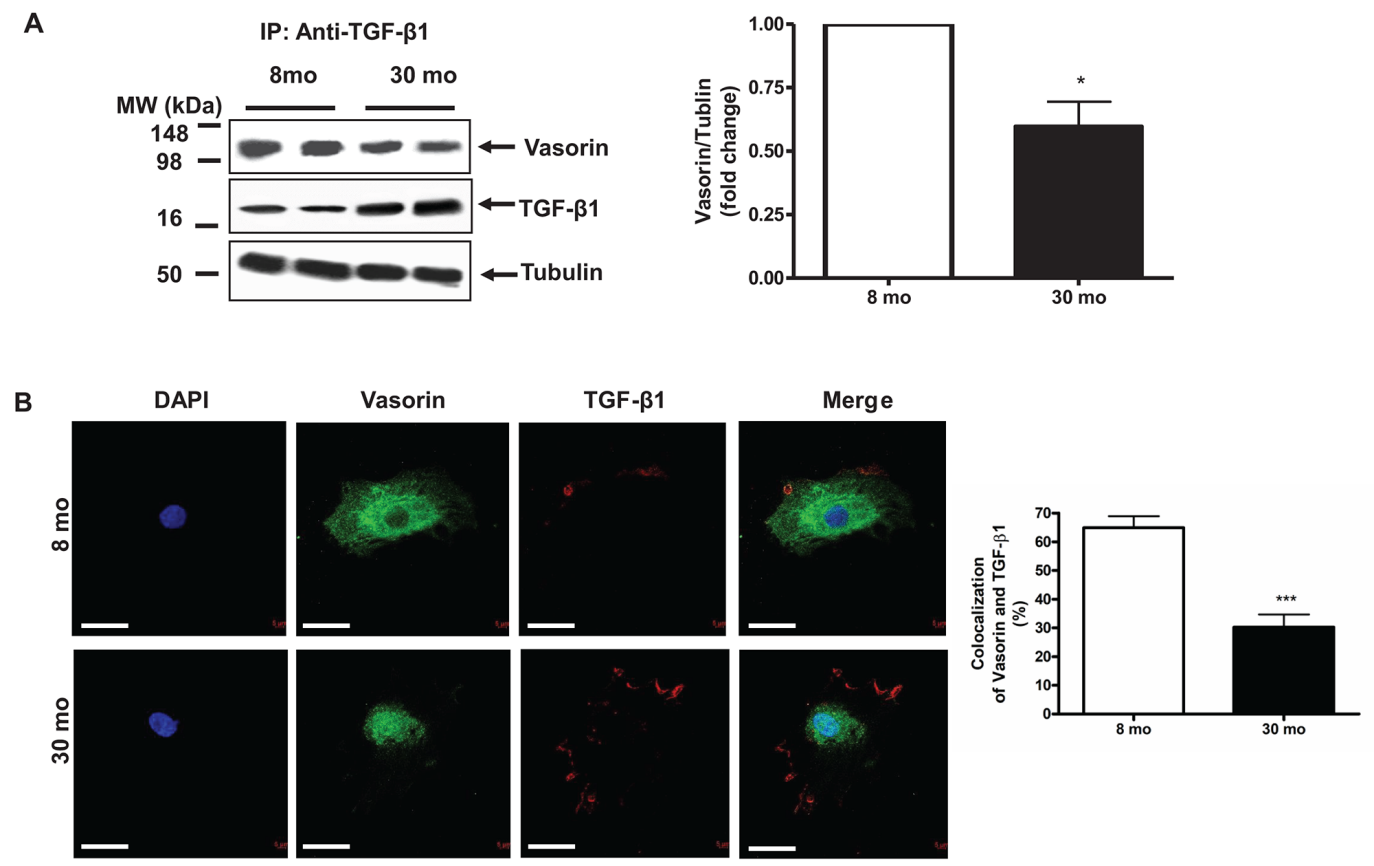
The Interaction of vasorin and TGF-β1 in VSMCs with aging **(A)** Co-immunoprecipitation of TGF-β1 and vasorin in VSMCs. Cell lysates were immunoprecipitated with an anti- TGF-β1 antibody and then the western blotting against both vasorin and TGF-β1 were performed. Data (right panel) shown as mean ± SEM (n= 4 independent experiments/group). Each column represents a normalized ratio (fold-changes) to tubulin and to 8 mo. *T*-test, ^*^=p<0.05. **(B)** Dual labelling (X630) of vasorin (green) and TGF-β1 (red) in VSMCs. All micrographs were taken at the same exposure time. Data of the relative colocalized area (%, right panel) shown as mean ± SEM (n= 4 independent experiments/group). *T*-test, ^*^=p<0.05. *T*-test, ^***^=p<0.001. Bar scale, 10 μm.

It is known that vasorin effectively traps TGF-β1 protein and blocks its signaling in VSMCs [2]. In the current study, co-immunoprecipitation experiments demonstrated that the abundance of vasorin bound TGF-β1 significantly declined in old VSMCs when compared to those from young cells (Figure 5A). Accordingly, dual immuno-labeling indicated that the extent of co-localization of vasorin and TGF-β1 markedly declined in old VSMCs compared to young cells (Figure 5B).

**Figure 6:**
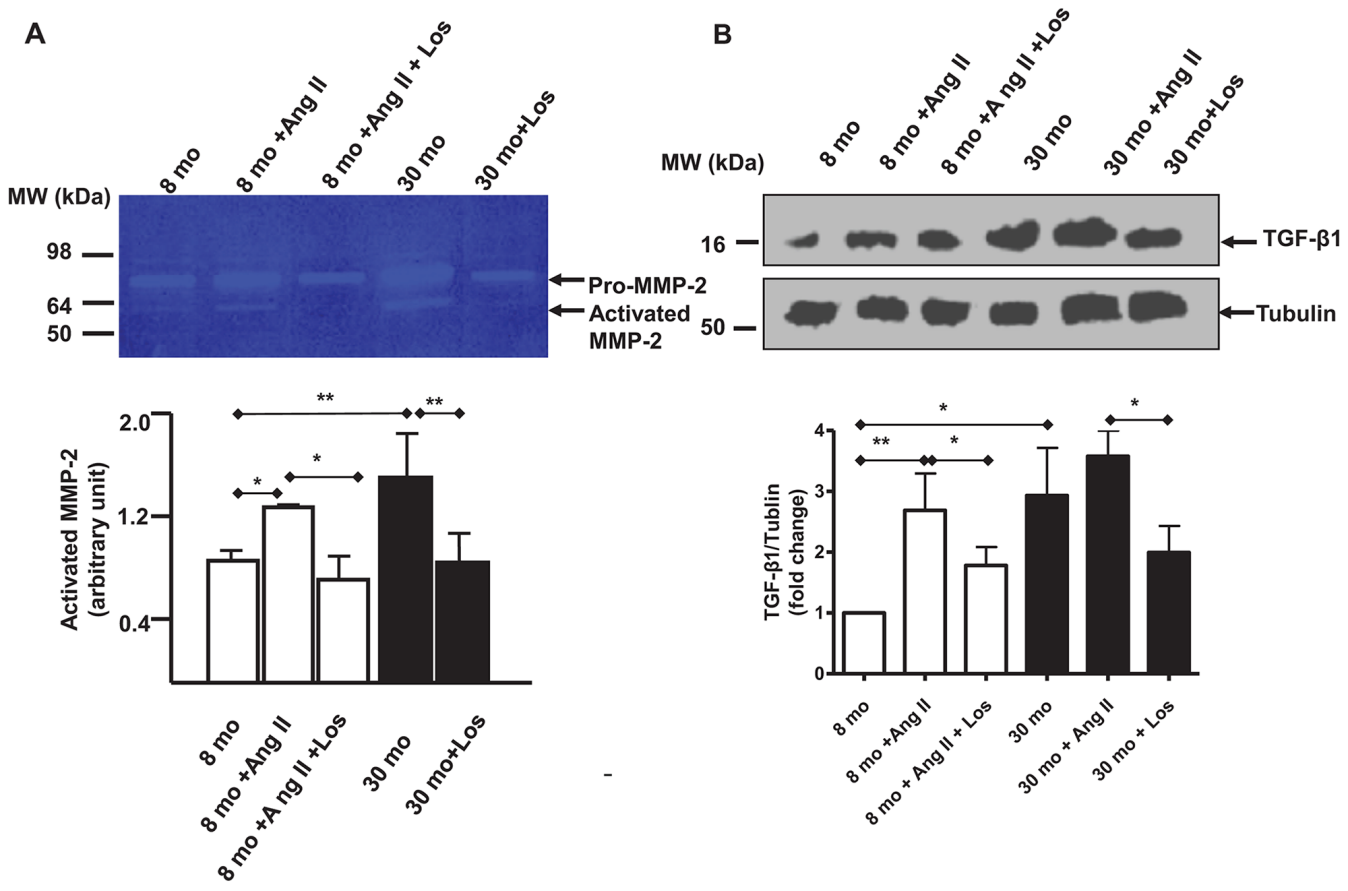
Ang II, MMP-2 and TGF-β1 activation in VSMCs **(A)** Representative zymogram of the medium from early passage cultured VSMCs treated with either Angiotensin II (Ang II) Losartan (Los) (upper panel). Cells have been pre-treated for 1 hour with Los (5X10^-6^ M), and then exposed for 24 hours to a medium containing both Ang II (5X10^-6^ M) and Los (5X10^-6^ M). Data (lower panel) shown as mean ± SEM (n=4 independent/group). One-way ANOVA followed Bonferroni post hoc test, ^*^=p<0.05, and ^**^=p<0.01. **(B)** Representative western blots of TGF-β1 from lysates from early passage cultured VSMCs treated with either Ang II or Los (upper panel). Cells have been pre-treated for 1 hour with Losartan (5X10^-6^ M), and then exposed for 24 hours to a medium containing both Ang II (5X10^-6^ M) and Los (5X10^-6^ M). Data (lower panel) shown as mean ± SEM (n=4 independent/group). Each column represents a normalized ratio (fold-changes) to tubulin and to untreated 8 mo VSMCs. One-way ANOVA followed Bonferroni post hoc test, ^*^=p<0.05, and ^**^=p<0.01.

To determine whether Ang II, via AT1 signaling, affects the activation of both MMP-2 and TGF-β1 in VSMCs with aging, young VSMCs were treated with Ang II and results showed that Ang II significantly elevated the levels of both activated MMP-2 and activated TGF-β1, resembling those of old untreated cells. However, these effects were substantially diminished by AT1 antagonist, Los (Figure 6A & 6B).

**Figure 7:**
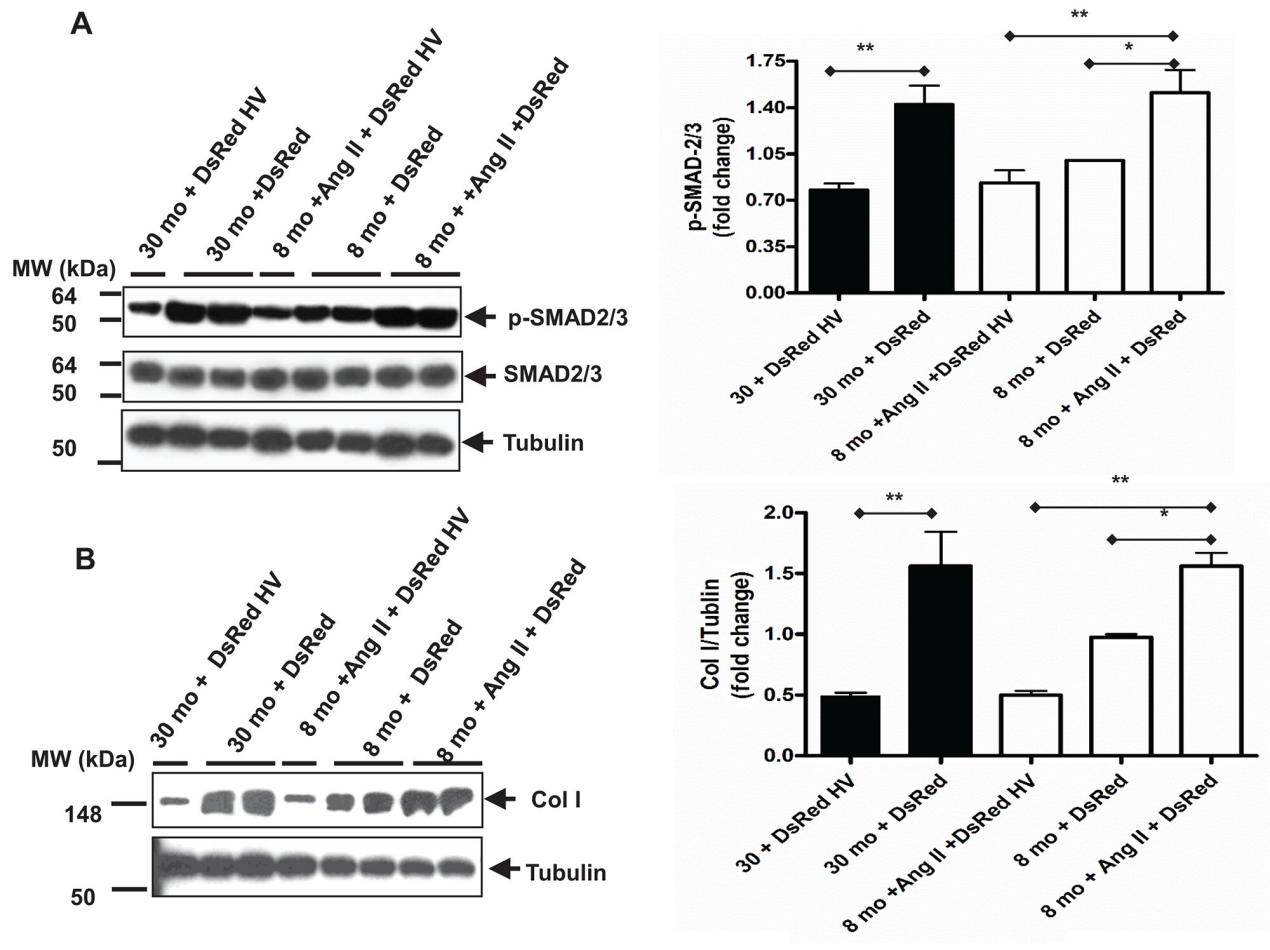
Age increases TGF-β1 downstream profibrotic molecules in VSMCs mediated by over-expressing vasorin **(A)** Representative western blots for p-SMAD-2/3 and total SMAD-2/3 of VSMCs transfected with either control DsRed or DsRed containing human vasorin cDNA (DsRed HV) plasmid and treated with or without Ang II (5X10^-6^ M) for 24 hours. Data (right panel) shown as mean ± SEM (n=6 independent/group). Each column represents a normalized ratio (fold-changes) to tubulin and to 8 mo + DsRed. One-way ANOVA followed Bonferroni post hoc test, ^*^=p<0.05, and ^**^=p<0.01. **(B)** Representative western blots for collagen Type I (Col I) of VSMCs transfected with either control DsRed or DsRed containing human vasorin cDNA (DsRed HV) plasmid and treated with or without Ang II (5X10^-6^ M) for 24 hours. Data (right panel) shown as mean ± SEM (n=6 independent/group). Each column represents a normalized ratio (fold-change) to tublin and to 8 mo + DsRed. One-way ANOVA followed Bonferroni post hoc test, ^*^=p<0.05, and ^**^=p<0.01.

Next, we wanted to investigate whether the interplay between vasorin and TGF-β1, like Los, block the signaling of Ang II / TGF-β1 in VSMCs with aging. To this end, vasorin was overexpressed in both young and old VSMCs by using a plasmid containing the human vasorin cDNA (a representative experiment of VSMCs overexpressing vasorin is reported in Supplementary Figure 3). Indeed, treating young VSMCs with Ang II increased the levels of TGF-β1 downstream molecules p-SMAD-2/3 and Col I, up to the levels of untreated old VSMCs, and this set of effects were abolished by the overexpression of vasorin (Figure 7A & 7B).

**Figure 8:**
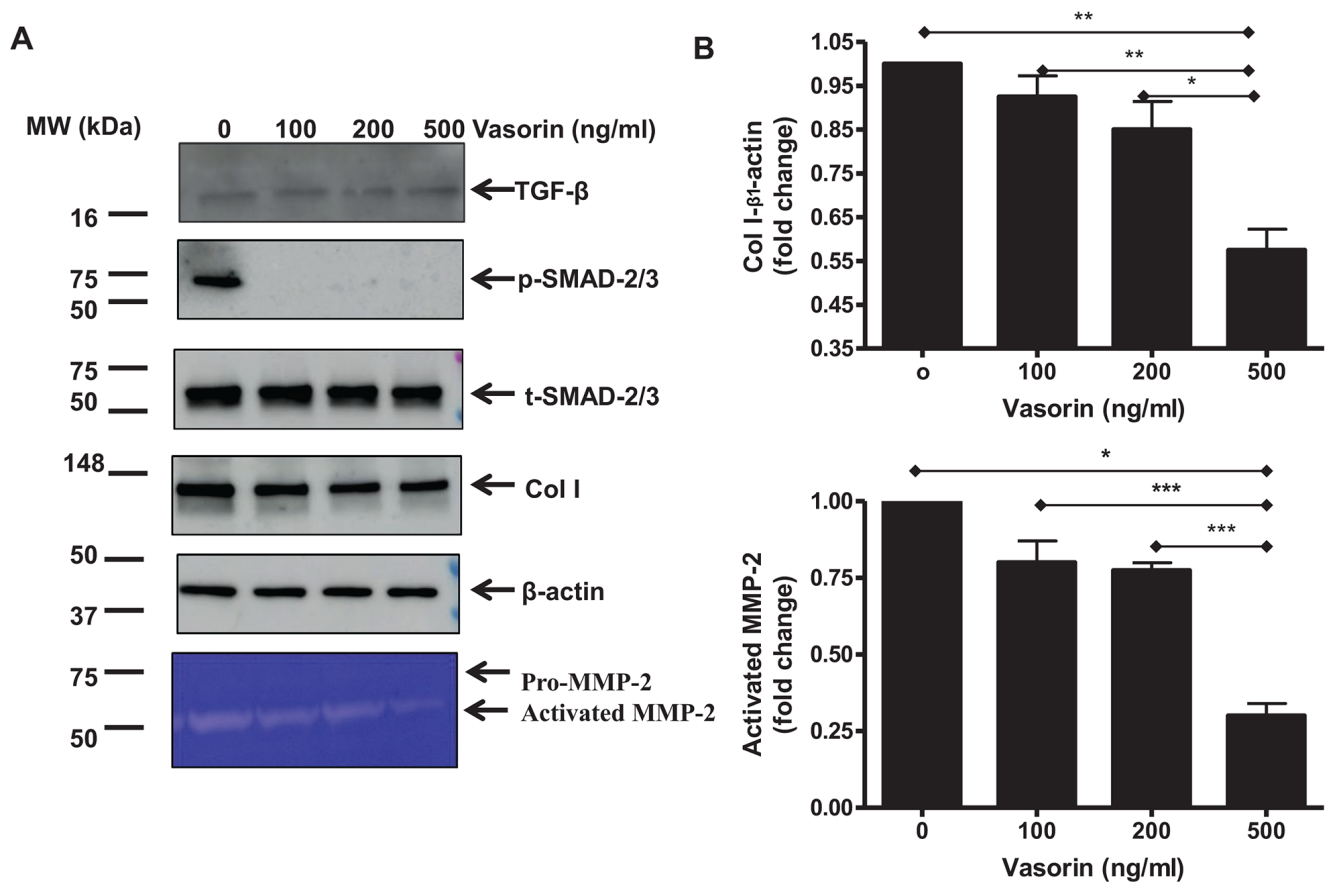
TGF-β1 downstream profibrotic effects in old VSMCs regulated by recombinant human vasorin proteins **(A)** Representative western blots of TGF-β1, p-SMAD-2/3, SMAD-2/3, Coll I, β-actin, and Zymogram from lysates of early passage cultured VSMCs treated with or without recombinant human vasorin proteins at the indicated doses. **(B)** Data shown as mean ± SEM (n=3 independent/group). Each column represents a normalized ratio (fold-change) to β-actin and to untreated control (0). One-way ANOVA followed Bonferroni post hoc test, ^*^=p<0.05, ^**^=p<0.01 and ^***^=p<0.001.

Finally, we ran a set of experiments to further investigate whether the above effects are linked to variations of the active TGF-β1 levels, or to the alleviation of its receptor signaling. Results shown that treating old VSMCs with the recombinant human vasorin protein did not affect the level of activated TGF-β1 (Figure 8, top panel), but markedly blocked the TGF-β1 downstream signals dose-dependently inhibiting the expression of p-SMAD/-2/3, Coll I, and activated MMP-2 (Figure 8). These findings suggest that vasorin alleviates the fibrogenic signaling in VSMCs by limiting the TGF receptor downstream signaling independently from the levels of activated TGF-β1.

Taken together, the current findings further prove that vasorin directly binds to TGF-β1 and modulates its signaling in VSMCs with aging.

### Vasorin, Ang II associated MMP-2 activation, and VSMC invasion

**Figure 9:**
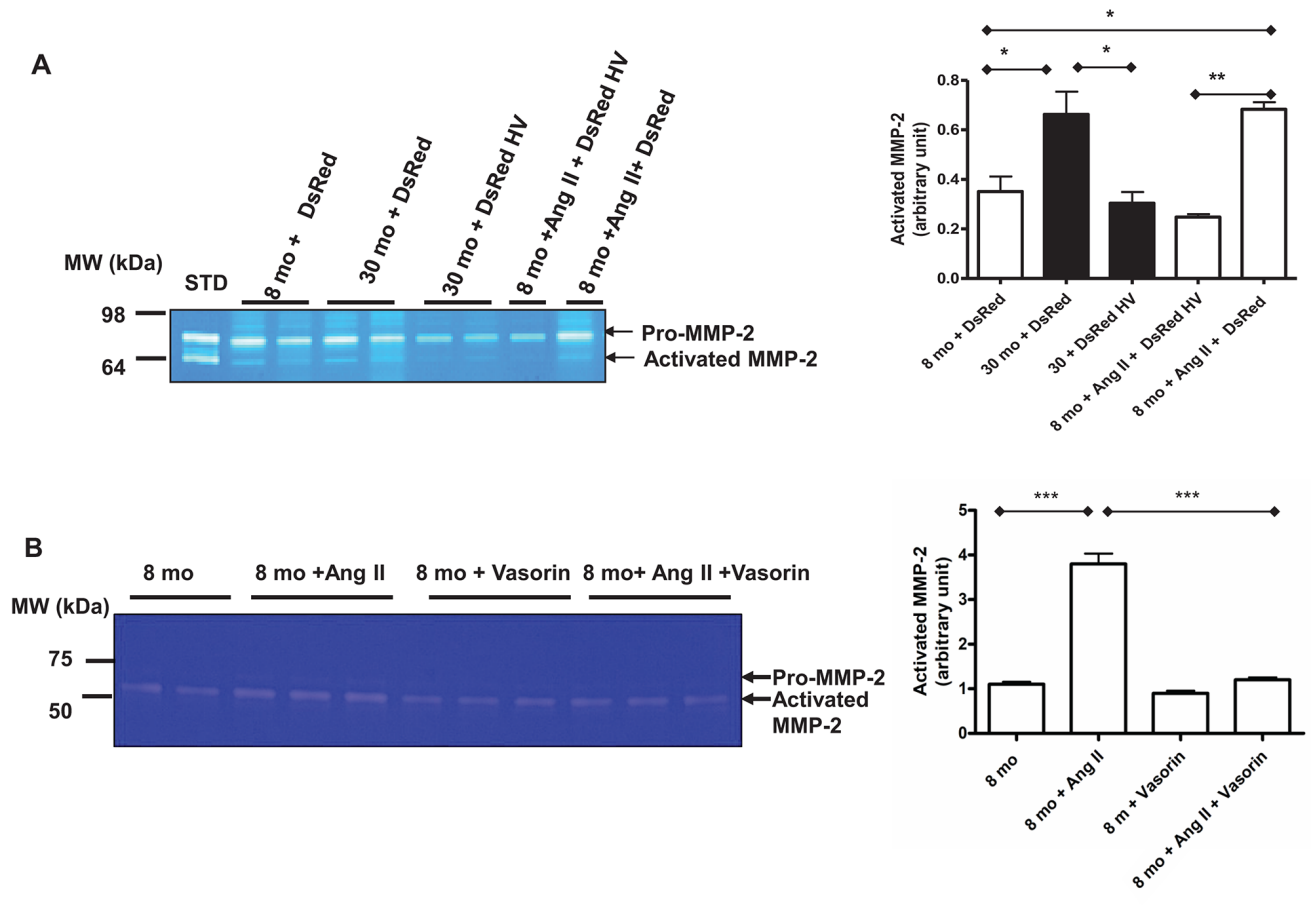
The Ang II associated MMP-2 activation with aging mediated by vasorin **(A)** Representative zymogram from VSMCs transfected with either control DsRed or DsRed containing human vasorin cDNA (DsRed HV) plasmid and treated with or without Ang II (5X10-^6^M) for 24 hours. Data (right panel) shown as mean ± SEM (n=4 independent experiments/group). One-way ANOVA followed Bonferroni post hoc test, ^*^=p<0.05 and ^**^=p<0.01. STD=standard: activated MMP2 protein. **(B)** Representative zymogram from VSMCs treated with Ang II (5X10-6M) with or without recombinant human vasorin protein (100 ng/ml). Data (right panel) shown as mean ± SEM (n=3 independent experiments/group). One-way ANOVA followed Bonferroni post hoc test, ^***^=p<0.001.

MMP-2 activation and its subsequent driving of VSMCs invasion exert an important role in relaying the signaling of Ang II in adverse arterial remodeling. Here, we sought to demonstrate that vasorin overexpression can affect Ang II associated MMP-2 activation and VSMC invasion. Indeed, exposure of young VSMCs to Ang II increased the levels of activated MMP-2 to those of untreated old VSMCs, and these effects were substantially inhibited by the overexpressing of vasorin (Figure 9A) and by the treatment with the recombinant human vasorin protein (Figure 9B). Moreover, old VSMCs that were transfected with the vasorin plasmid showed reduced levels of MMP-2 activation, comparable with levels of untreated young VSMCs (Figure 9A).

Together these results support the hypothesis that vasorin, like Ang II blockade, modifies Ang II associated MMP-2 activation in VSMCs with aging.

**Figure 10:**
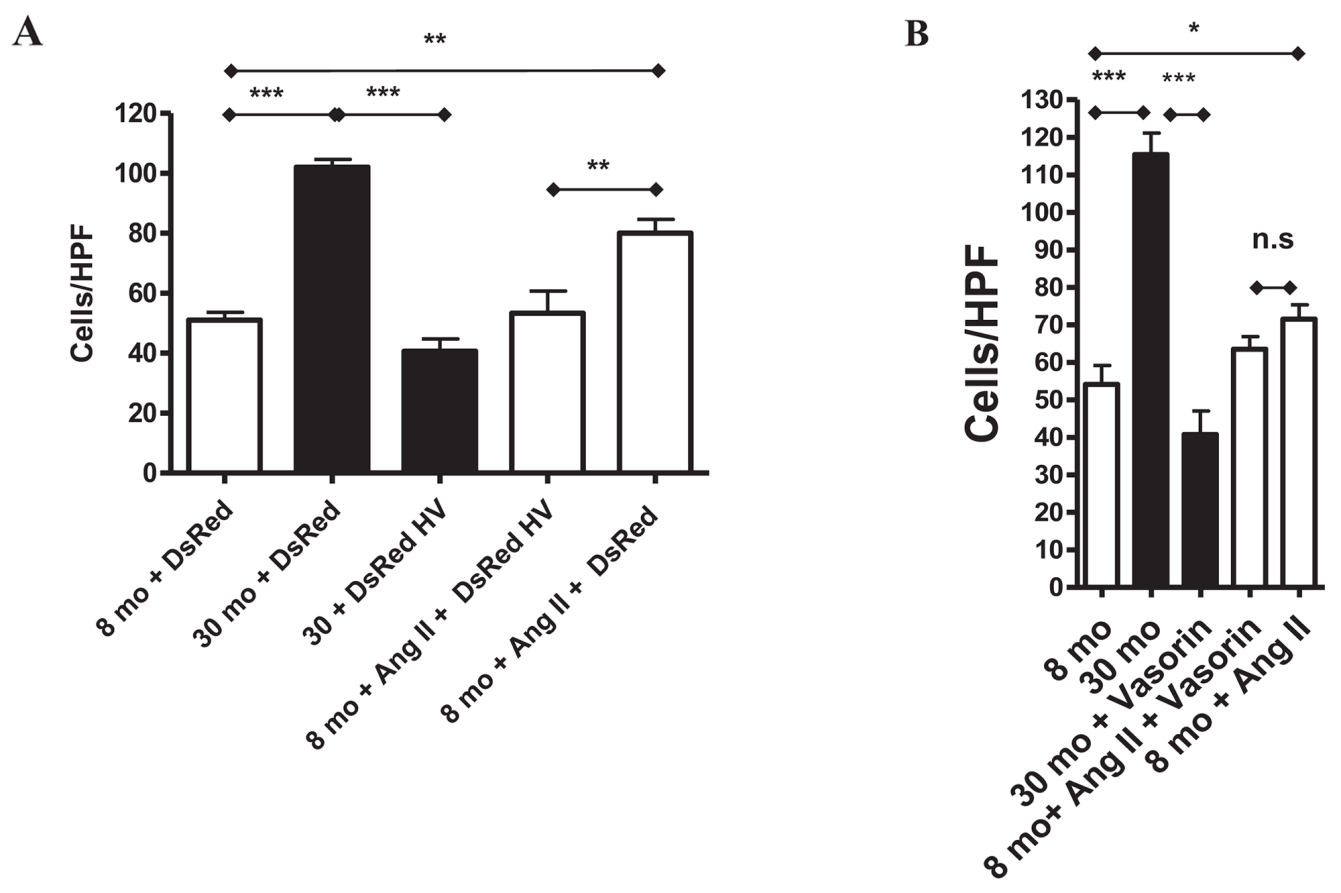
Vasorin decreases both Ang II- and aging-associated phenotypic shift of VSMCs into invasiveness **(A)** Invasion assay of VSMCs transfected with either control DsRed or DsRed containing human vasorin cDNA (DsRed HV) plasmid and treated with or without Ang II (5X10^-6^ M) for 4 hours using modified Boyden chamber. Data shown as mean ± SEM (n=3 independent experiments/group). One-way ANOVA followed Bonferroni post hoc test, ^**^=p<0.01 and ^**^=p<0.001. HPF= high power field. **(B)** Invasion assay of VSMCs treated with without Ang II and human recombinant vasorin protein for 4 hours using modified Boyden chamber. Data shown as mean ± SEM (n=3 independent experiments/group). One-way ANOVA followed Bonferroni post hoc test, ^*^=p<0.5 and ^**^p<0.001. HPF= high power field.

Finally, we wanted to test whether vasorin inhibits the Ang II associated VSMC invasion. Indeed, young Ang II-treated VSMCs showed a significant increase in their invasive capacity (similar to that of old cells), which was abolished by both vasorin overexpression and recombinant human vasorin protein treatment (Figure 10A and 10B).

Taken together, these results indicate that vasorin may modify Ang II associated MMP-2 activation and VSMC invasion.

## DISCUSSION

**Figure 11:**
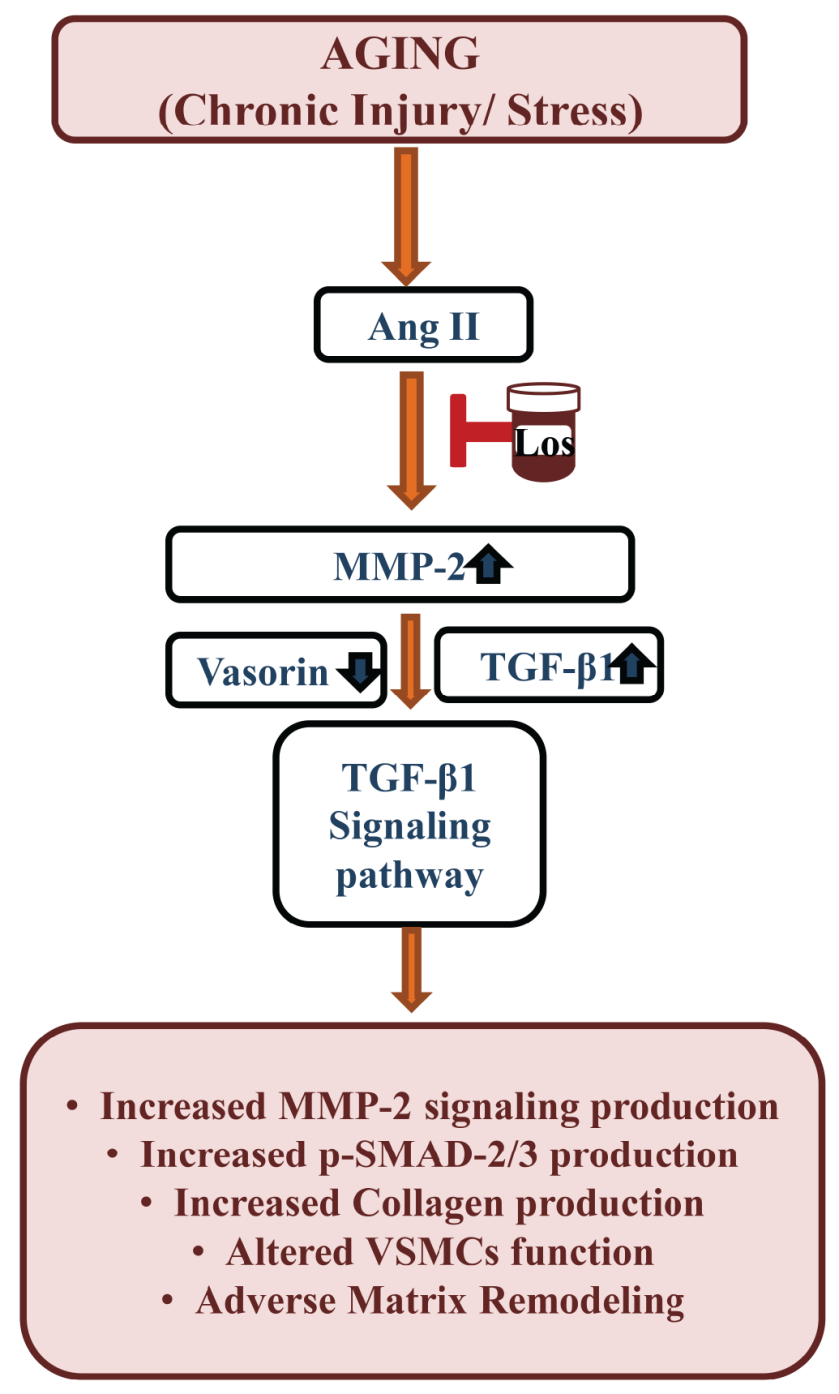
Schematic diagram showing the Ang II-associated TGF-β1 signaling affected by vasorin cleavage due to MMP-2 activation in VSMCs or arterial walls with aging Ang II=angiotensin II, Los=losartan, MMP-2=matrix metalloproteinase type II, TGF-β1=tissue transforming growth factor-beta1, VSMC=vascular smooth muscle cells.

The present study, for the first time, demonstrate that a reduction in the glycoprotein vasorin, coincides with the increases in Ang II signaling, MMP-2 and TGF-β1 activation, in the arterial wall or VSMCs via AT1 signaling with advancing age (Figure 11). The reduced vasorin protein in the aging arterial wall or cells is due mainly to an increase of vasorin cleavage by MMP-2. A reduction in the vasorin protein amplifies the Ang II signaling, MMP-2, and TGF-β1 activation in the arterial wall or cells with aging (Figure 11). Thus, preserving an effective level of intact vasorin is a novel molecular target to maintain VSMC quiescence and collagen homeostasis in the arterial wall that accompanies advancing age.

The decrease of vasorin in the arterial wall or VSMCs with aging is mediated by activation of the Ang II/AT1/MMP-2 signaling cascade. The current study, for the first-time, documents that similar to the aging process, Ang II decreases the expression of vasorin protein in the arterial wall or VSMCs via an activation of the AT1 receptor; in contrast, a blockade of the AT1 receptor signaling increases the expression of vasorin in VSMCs. Ang II does not markedly alter the mRNA levels of vasorin in VSMCs. These findings suggest that the age associated decreases in vasorin protein abundance is due at least partially to its post-translational modifications through cleavage. Prior studies and current findings suggest that the vasorin protein is susceptible to be degraded by activated MMP-2 [17, 18]. It is well-known that Ang II or aging increases the activation of MMP-2 in the arterial wall, arterial rings, and VSMCs [20–23]. The current findings show that the activated MMP-2 substantially degrades the vasorin protein *in vitro* and *in vivo*. Importantly, the prevention of vasorin cleavage by the MMP inhibitor PD166793, is linked with the retardation of adverse age-associated arterial fibrogenic remodeling [24]. Notably, the current study indicates that the aging process itself does not alter the expression of the ADAM17 protein in VSMCs, which is known to cleave vasorin *in vivo* and *in vitro* [16, 19–22], thus excluding this proteinase from the current analysis of aging-related phenomena. Taken together these findings suggest that MMP-2 plays an important contributory role in decreasing vasorin protein in the arterial wall or VSMCs with advancing age.

Vasorin, interacting with TGF-β1, in a manner that resembles the Ang II receptor antagonist, decreases pro-fibrogenic effects in aging VSMCs. Indeed, vasorin strongly binds to TGF-β1, and subsequently blocks TGF-β receptors type I and II activation and their downstream signals [20, 25]. Overexpression of vasorin in old VSMCs decreases TGF-β1 downstream signaling (p-SMAD-2/3 and Col I production) to levels resembling those achieved by Losartan-treated old cells. Further, in young VSMCs, overexpression of vasorin counteracts the Ang II-induced increases in TGF-β1 signaling (p-SMAD-2/3 and Col I production). Importantly, old VSMCs treated with the vasorin protein, substantially blocked the expression of TGF-β1 downstream molecules such as p-SMAD-2/3, although the levels of activated TGF-β1 remained constant, further supporting the hypothesis that vasorin alleviate TGF-β1 signaling by blocking its access to the TGF-β receptors. These findings also further support the novel concept that vasorin physically traps TGF-β1, mimicking the AT1 antagonist Los, thus mitigating Ang II associated fibrotic effects in the arterial wall or VSMCs with aging.

Increased intimal VSMCs infiltration is a key cellular event for arterial remodeling with aging and is closely associated with the activation of Ang II associated MMP-2 [4, 26]. Ang II increases MMP-2 activation and promotes a rapid transition from a contractile differentiated phenotype to a synthetic dedifferentiated invasive phenotype of VSMCs [4]. Vasorin overexpression or protein treatment inhibits Ang II associated activation of MMP-2 and the invasiveness of VSMCs with aging. These findings reveal that vasorin modulates aging/Ang II associated activation of MMP-2 and VSMCs invasiveness.

Growing evidence indicates that vasorin is involved in the modulation of adverse arterial remodeling such as arterial restenosis and calcification [2, 3, 27]. Local vasorin protein levels are inversely related to the degree of arterial restenosis [3]. Reduction of circulating vasorin is closely correlated with the incidence and severity of aortic valve calcification in humans [27]. Conversely, overexpression of vasorin directly alleviates neointima thickening [2]. Administration of a novel immunosuppressant FK778 or inhibition of miR-146a significantly reduces coronary restenosis and arterial neointima formation, a phenomena that is accompanied by the upregulation of vasorin levels [23, 28–32].

Taken together, these data indicate that vasorin is not only involved in acute arterial injury, but also in age-associated vascular remodeling through the modulation of the Ang II signaling cascade. Vasorin levels are reduced by age-associated increases in Ang II due mainly to its cleavage by MMP-2. A reduction in vasorin magnifies the Ang II mediated increase in the TGF-β1 signaling cascade and invasion of VSMCs with aging. Thus, maintaining the levels of arterial vasorin is a potential novel therapeutic platform to prevent age-related adverse vascular remodeling.

## MATERIALS AND METHODS

### Experimental animals

All procedures were performed per protocols approved by the National Institute on Aging (NIA) in accordance with the National Institute of Health (NIH) Animal Care and Use Committee. Eight-month-old (8mo, young) and 30-month-old (30mo, old) male Fisher 344 crossbred Brown Norway (FXBN) rats were obtained from National Institution on Aging Aged Rodent Colonies. Animals were sacrificed and thoracic aortae were harvested as previously described [33]. Aortic tissues were paraffin embedded and sectioned for histology study and their protein and ribonucleic acid (RNA) were extracted for western blotting and Real-time polymerase chain reaction (RT-PCR) analysis.

### VSMCs in culture

VSMCs were isolated from both young and old FXBN rat aortae as previously described [34]. Cells were sub-cultured (passage 3-6) in modified DMEM with 10% fetal bovine serum (FBS). After serum removal for 24 hours, cells were treated with Ang II (Sigma) or Losartan (Toronto Research Chemicals, E96000) or human vasorin protein (Sino Biological Inc., Beijing, China) or vehicle in 2.5% FBS for 24 hours. Other treatments are reported in the figure legends. Cultured VSMCs were lysed for protein and RNA isolation for Western Blot and q-RT-PCR analysis.

### Immunostaining, immunofluorescence staining, and morphometric analysis

Staining of aortic walls and VSMCs were performed as described in previous studies [35]. For immnocytostaining, VSMC were fixed and processed for immunofluorescence with anti-vasorin and anti-TGF-β1 antibodies. Images were taken using the confocal espectral Olympus FV1000 (Olympus, Center Valley, PA, USA). Details of primary antibodies used are listed in Table 1. The ratios of target immunohistochemical or immunofluorescence staining positive area to the total tissue or cell area were determined via computer-imaging program according to the instruction provided by manufacture utilizing light microscopy (MetaMorph Imaging System, Universal Imaging Corp., PA).

**Table 1:** Primary Antibodies

Antibody	Specie	Titer Blotting	Titer Staining	Source
α-SMA	M	1:1000		Sigma, MO
ADAM17	R	1:1000		Abcam, MA
β-actin	M	1:5000		Sigma, MO
Col I	R	1:500	1:80	Santa Cruz, CA
GAPDH	M	1:4000	1:50	Sigma, MO
SMAD 2/3	R	1:200	1:50	Santa Cruz, CA
TGF-β1	R	1:500	1:100	Santa Cruz, CA
Tubulin	M	1:1000		Santa Cruz, CA
Vasorin	R	1:500	1:50	Santa Cruz, CA

R=rabbit; and M=mouse.

### Real-time quantitative reverse transcription PCR

Real-Time Quantitative Reverse Transcription PCR (q-RT-PCR) of rat aortic vasorin was performed according to modified methods as previously described [26, 34]. Vasorin primer information: CTCA GCCACAGTCGTCTCC for the forward; and GA TGGGCAGCTCTGTACTCC for the reverse. In brief, RNA was extracted from frozen aortae or cultured VSMCs using Trizol reagent (Thermo Fisher Scientific, Halethorpe, MD). Real-time PCR was performed per the SYBRGreen PCR protocol (Applied Biosystems, Foster City CA). Each sample was tested in quadruplicate. Data are expressed using the formula: quantity=10^–(Ct-Y intercept)/slope value)^ where Ct represents the threshold cycle value.

### Western blotting

Western blots of aortic tissue protein, and cultured VSMC protein were performed as previously described [26, 34, 35]. In brief, the total protein was quantified using the Bio-Rad (Hercules, CA, USA) according to the manufacturer’s instructions. In breif, 10 μg of total proteins were run on 4-12% NuPAGE gels (Thermo Fisher Scientific, Halethorpe, MD), transferred to a PVDF membrane and immunoblotted with antibodies against vasorin, ADAM17, p-SMAD-2/3, SMAD-2/3, Collagen I (Coll I), α-tubulin, and β-actin with the corresponding horseradish peroxidase-conjugated secondary antibodies and detected with ECL. Details of the primary antibodies used are listed in Table 1.

### PAGE zymography

MMP-2 activity was determined by PAGE zymography based on prior reports [36]. Cell lysates (10μg) were added to nonreducing sample buffer (1:3, v/v) and loaded on to Novex 10% zymogram plus (gelatin) gels (Thermo Fisher Scientific, Halethorpe, MD); electrophoresis was performed at 100 V for ∼2 h at 4 °C. The gels were then rinsed trice with 2.5% Triton X-100 to remove SDS and renature the MMPs in renaturing buffer. After a brief wash in distilled water, gels were incubated overnight at 37 °C in developing buffer with gentle agitation. The gels were stained with Coomassie® Brillant Blue R-250 (Sigma), and MMP activities were detected as transparent bands on the blue background.

### Co-immunoprecipitation

Co-immuno-precipitation was performed as described previously [37]. VSMCs from young rat aortae were lysed with non-denaturing lysis buffer (20 mM Tris HCl, 150 mM NaCl, 10% glycerol, 1% glycerol, 1% Nonidet P-40, and 2mM EDTA). After that, 100 μl of Protein A/G plus bead slurry were added to 400 μg of precleared lysate, incubated for 1 hour at 4°C with gentle agitation, and then centrifuged at 14,000 x g for 15 minutes at 4°C. The supernatants were transferred to a fresh tube, to which 40 μl of TGF-β1 antibody was added, then samples were further incubated at 4°C for 3 hours. To each tube, 100 μl of protein A/G plus bead slurry was added and incubated at 4°C overnight. The beads were collected by centrifugation and washed three times with lysis buffer. All supernatant was removed and 50 μl of 2x Laemmli sample buffer was added before heating at 95°C for 5 minutes. The samples were separated with 4% to 12% NuPAGE gels and immunoblotted with antibodies against vasorin and TGF-β1.

### Overexpression of vasorin

Overexpression of the vasorin gene was accomplished via a transient transfection of rat VSMCs with a bicistronic expression vector, pIRES2-DsRed (Clontech Laboratories, Inc. CA; Catalog No. 632540), containing human vasorin cDNA (HV-cDNA). The HV-cDNA, containing a C-terminus tag, kindly provided by Dr Yuichi Iked (University of Tokyo) was created through standard PCR procedures using a recombinant adenovirus as a template (Adeno-X system by Clontech). The transfection plasmid, IRES-DsRed, was obtained by cloning the PCR amplification product into the pIRES2-DsRed bicistronic expression vector (Clontech, CA) using the *EcoRI* and *BamHI* sites. The primer sequences used to create the PCR fragment with the two restriction sites were, 5’-CTGAATTCATGTGCTCCAGGGTCCCT-3’ for the forward and 5’-TAGGATCCTCGGATCATCCAGCACAGT-3’ for the reverse. The sequences were confirmed, and the PCR fragment was cloned into the pIRES2-DsRed bicistronic expression vector (Clontech, CA). Plasmid transfection of young and old rat VSMCs was achieved using the Fugene-HD transfection reagent following the manufacturer’s protocol (Roche Diagonostics, Mannheim, Germany). After 48 hours, cells were sorted with flow cytometric analysis to positively select the red fluorescent cells to obtain a pure population (over 90%) of overexpressing vasorin cells.

### VSMC invasion

Invasion assays were performed on young and old VSMCs using a modified Boyden chamber with a Matrigel (cat #356234, BD Bioscience-Discovery) coated filter (Neuroprobe) as previously described [36]. Young VSMCs overexpressing vasorin were treated with or without Ang II (100 nmol) for 24 hours. PDGF-BB (10 ng/ml) served as a chemoattractant control. Addition of Matrigel to the filter of the modified Boyden chamber, mimics the basement membrane of cells, requiring cells to release MMP-2 to degrade the membrane before invading through the pore towards the chemoattractant.

### Cleavage of the recombinant human vasorin and monkey arterial lysate containing vasorin

Vasorin cleavage was performed using a modified protocol as previously described [38]. Purified recombinant human MMP-2 (R&D Systems), recombinant vasorin (Sino Biological Inc.) and its inhibitor GM 6001 (abcam), homogenous monkey aortic lysate (grantome.com/grant/NIH/ZIA-AG000238-05) were dissolved in 50 mM Tris buffer containing 5mM CaCl_2_ and 0.2M MgCl_2_, pH 7.5. MMP-2 (50nM) was added to the vasorin (400 ng/ml)/aortic lysate (25 μg/ml) with or without GM 6001 (20nM) and incubated in the incubation buffer (pH7.5) containing 1% Triton X-100, 50 mM Tris-HCI, 5mM CaCI_2_ 1μM ZnCI_2_ at 37 °C for 24 h. The aliquots (20 μl) were withdrawn to 8 μl of 1.0 m EDTA and then mixed with the 5x sample buffer (60 mm Tris-HCl, pH 6.8, 2% SDS, 10% glycerol, 5% β-mercaptoethanol, 0.01% bromophenol blue) and subjected to SDS-PAGE followed by Western immunoblotting.

### Statistical analysis

All experiments were performed in at the least three independent experiments and three different rats. Data analysis was performed using GraphPad Prism. Data were expressed as Mean ± SEM. For two groups, comparisons of means were made using Student’s unpaired two tailed test (*T*-test), and for more than two groups, comparisons of means were made using one-way ANOVA with Bonferroni post hoc test. p<0.05 was considered as significant level.

## SUPPLEMENTARY MATERIALS FIGURES


