# Supplementary material for: Reduced vasorin enhances angiotensin II signaling within the aging arterial wall
Source: Oncotarget. 2018 Jun 5;9(43):27117–32. doi: 10.18632/oncotarget.25499 (PMC6007470; doi:10.18632/oncotarget.25499)
Supplement: Supplementary file 1 [file oncotarget-09-27117-s001.pdf]

Reduced vasorin enhances angiotensin II signaling within the aging arterial wall

SUPPLEMENTARY MATERIALS

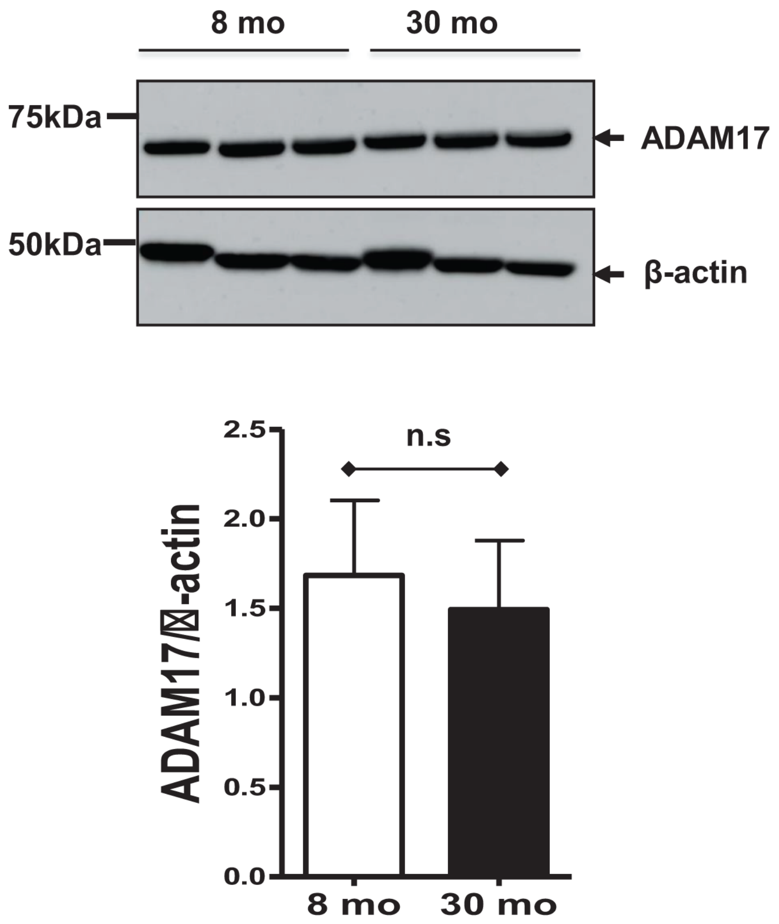

Supplementary Figure 1: Effects of aging on ADAM17 expression in VSMCs. Western blots of VSMC lysates for ADAM17. Data shown as mean ± SEM (n=3 independent experiments from n=3 rats/group. *T*-test, N.S, no significant.

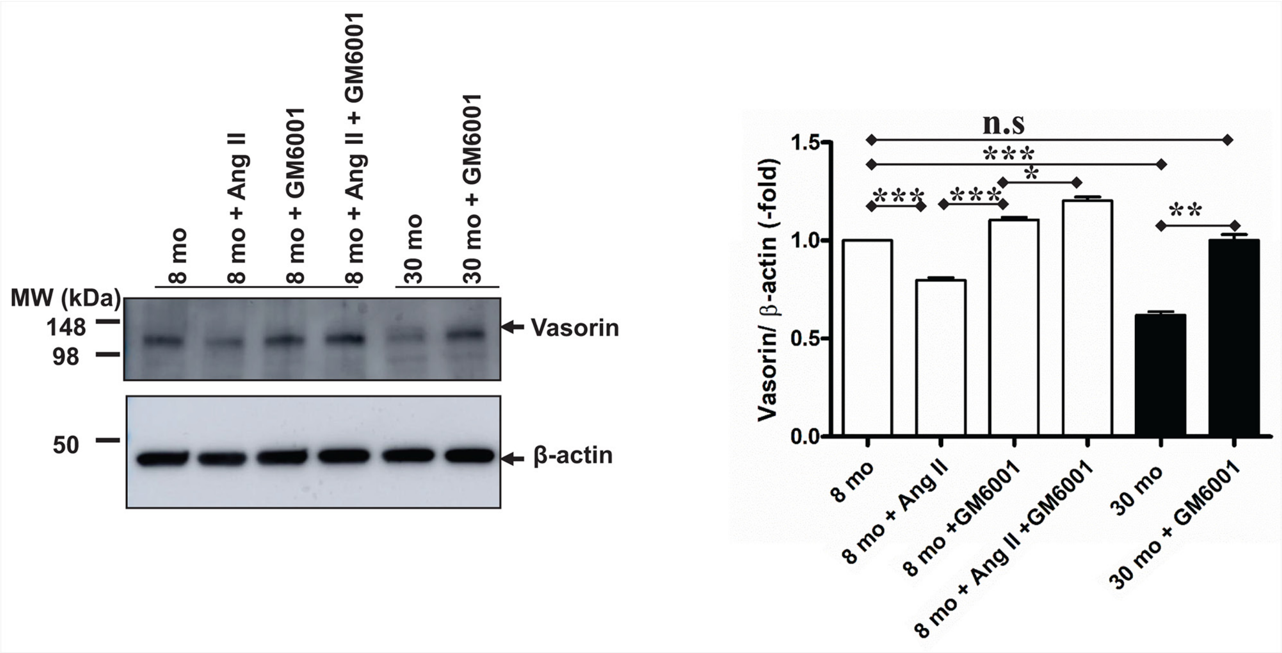

Supplementary Figure 2: MMP inhibition restores Ang II/aging induced the downregulation of vasorin protein in VSMCs. Representative western blots of VSMC vasorin. Cells were exposed for 24 hours to a medium containing Ang II (100 nM or GM6001 (20 nM) or Ang II plus GM6001. Data (right panel) shown as mean ± SEM (n=3 independent experiments from n=3 rats/ age group). One-way ANOVA followed Bonferroni post hoc test, *p<0.05, **=p<0.01; and ***=p<0.001.

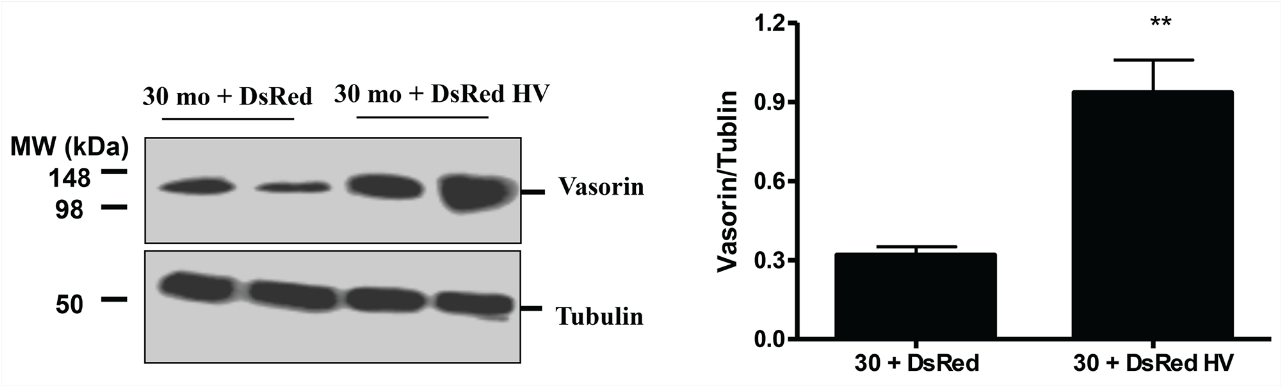

Supplementary Figure 3: Overexpression of vasorin in VSMCs. Representative western blots of old VSMC lysates for vasorin (left panel) and data shown as mean ± SEM (n=4 independent experiments, right panel). transfected with either control DsRed or DsRed containing human vasorin cDNA (DsRed HV) plasmid. *T*-test, **=p<0.001.
